# Structural, Psychological and Contextual Predictors of Car Use

**DOI:** 10.3389/fpsyg.2021.692435

**Published:** 2021-11-30

**Authors:** Alin Semenescu, Alin Gavreliuc

**Affiliations:** Department of Psychology, West University of Timișoara, Timișoara, Romania

**Keywords:** car use, sustainable transportation, psychological predictors, structural predictors, contextual predictors

## Abstract

Besides its undeniable advantages, personal car use generates a wide array of problems, among which its contribution to global warming is probably the most severe. To implement sound policies that are effective in reducing private car use, it is essential to first understand its important antecedents. Structural, psychological and contextual predictors were extensively studied independently, yet integrative approaches that investigate all these factors in a single theoretical model are lacking. The present study contributes to a more comprehensive understanding of car use behavior by proposing a model that includes structural, psychological and contextual determinants and tests this model on an international sample of drivers (*N* = 414). Responses were analyzed using a structural equation modeling approach. Results show that car use habits, perceived behavioral control, policy measures, fuel cost, infrastructure, temperature and level of precipitations significantly influence car use behavior. Such results support the inclusion of both structural (i.e., hard) and psychological (i.e., soft) factors in the design of policy interventions, while also considering contextual situations. Implications for policy and practice are discussed.

## Introduction

Undeniably, road transportation serves many personal and societal needs, yet it also generates a wide array of problems. Among the most acute are air pollution and global warming, to which the transportation sector remains a significant contributor. It is responsible for about a fifth of all carbon dioxide (CO_2_) emitted annually into the atmosphere (Bamberg and Rees, 2017). Furthermore, while other sectors are gradually decreasing their contribution to climate change, the contribution of the transportation sector continues to rise (Friman et al., 2013), which places it at the frontline of the fight against global warming. Unquestionably, technological advances alone cannot be a panacea for all the negative effects of car transportation; to reach sustainable mobility, policymakers must focus also on car demand reduction (Graham-Rowe et al., 2011; Richter et al., 2011). As a consequence, a series of measures to decrease car use, commonly known as travel demand management (TDM; Loukopoulos, 2007), were applied. TDM measures are generally classified into two categories: Measures aiming to modify travel behavior by modifying social conditions or structures are called *structural* measures, while those aiming to modify people’s perceptions, beliefs, attitudes, values or norms are the so-called *psychological* measures (Steg, 2003; Bamberg et al., 2011). These two types of measures change important structural and psychological determinants (e.g., individual variables, travel infrastructure, economic disincentives, etc.) in order to modify travel behavior. Aside from these two types of factors, research shows that people’s mobility patterns are influenced also by *contextual* (e.g., weather; see Böcker et al., 2013) and *demographic* factors (e.g., income, gender or age; see Giuliano and Dargay, 2006), introducing even more complexity into the picture (e.g., Carrus et al., 2021).

To be able to implement sound policies that discourage excessive car use, it is essential to first understand the important antecedents of such behavior. Independently, structural, psychological and contextual predictors of car use were extensively studied in empirical studies. Nevertheless, explaining car use from a single perspective is simplistic, as no perspective can adequately represent the multi-determinate character of travel behavior. Moreover, because predictors often correlate to a large extent, studying them in isolation can even be misleading, as results might portray a picture in which behavioral predictors are more numerous and effects are larger than when the same predictors are studied in larger models. Creating integrative models that account for multiple influences is therefore important, both for understanding the real complexity of car use behavior as well as for adequately informing future interventions aimed at reducing it. Nevertheless, integrative approaches were relatively few and limited in their range. For example, within the domain of psychological predictors, Liu et al. (2017) integrated the theory of planned behavior and the norm activation model in explaining intentions to reduce car use. In a more complex model, Klöckner and Blöbaum (2010), integrated both psychological predictors as well as objective constraints in explaining car use, while Noblet et al. (2014) employed a model that contained both structural and psychological predictors for explaining car travel reduction. However, to the knowledge of the authors, there were no attempts to propose models that integrate a comprehensive set of determinants. The aim of the present study is to contribute to a more comprehensive understanding of car travel behavior by proposing an integrative model that contains structural, psychological and contextual determinants of car use, while also controlling for relevant demographic variables. This approach will allow the possibility of answering two important questions: (1) How do these factors interrelate with one another in predicting car use, and (2) which of them exert the largest predictive effects?

### Previous Research

#### Structural Factors

Structural measures, colloquially known also as *hard* measures (Bamberg et al., 2011), are focused on changing travel behavior by modifying the physical environment (e.g., improving infrastructure) or by changing legal or economic policies (e.g., prohibiting car traffic in city centers, parking control, higher taxes on fuel, etc.). Evaluation studies conducted to examine the behavioral responses to such measures provided evidence that structural measures were often effective in decreasing car use. For example Jakobsson et al. (2002) showed that *economic policies* reduced driving for shopping trips, while Eriksson et al. (2010) found that people significantly expected to decrease their car use following a raised *price of fossil fuel*. Improvements in *travel infrastructure* such as upgrades in public transport service (e.g., Kristensen and Marshall, 1999; Cairns et al., 2004) or building new infrastructure (e.g., Arentze et al., 2001), have also revealed positive effects in reducing car use as well as in promoting more sustainable travel alternatives (see Pucher et al., 2010). For example, Dill and Carr (2003) found that for every additional mile of cycling lane per square mile there was an increase of about 1% in the proportion of commuters using the bicycle as transportation mode.

#### Psychological Factors

Structural measures are typically costly to implement and sometimes may even be politically unfeasible, as some measures (especially restrictive policies and disincentives) can be strongly opposed by the public. They have also been considered as insufficient for reducing car use (Stopher, 2004). Consequently, interest in *psychological* (or the so-called *“soft”*) measures has increased. Psychological measures are defined by Steg (2003, p. 190) as “strategies aimed at influencing people’s perceptions, beliefs, attitudes, values, and norms,” which are focused on changing travel behavior through voluntary, instead of coercive means. Allowing people the freedom to choose represents the primary reason why such measures are also better received by the public (Taylor and Ampt, 2003).

The implementation of soft measures is typically informed by theories, which specify the most important antecedents leading to behavioral change. Within the psychological paradigm, two theories were predominantly employed in transportation research to explain car use behavior, namely the *theory of planned behavior* (TPB; Ajzen, 1991) and the *norm activation model* (NAM; Schwartz, 1977). The TPB is a rational choice theory which stipulates that behavior is determined by behavioral intentions which, in turn, are influenced by *attitudes* (the degree of favorable or unfavorable rational evaluations of the behavior), *subjective norms* (perceived pressures from the social environment or significant others to behave in certain ways) and *perceived behavioral control* (the perceived easiness of performing the behavior). TPB model was useful in predicting travel mode choice in a considerable number of empirical studies (e.g., Heath and Gifford, 2002; Bamberg et al., 2003; Bamberg and Schmidt, 2003; Klöckner and Matthies, 2009), while two meta-analyses found that all TPB constructs significantly correlated with car use behavior (see Gardner and Abraham, 2008; Lanzini and Khan, 2017).

NAM, on the other hand, assumes that individual behavior is shaped by moral considerations. The model assumes that behavior is directly predicted by *personal norms*, which are defined as moral standards that people hold for themselves. According to the model, personal norms are activated only if one is aware of the consequences of her/his behavior (*awareness of consequences*) and feels a sense of responsibility for such consequences (*ascription of responsibility*). In transportation research, NAM was extensively used to predict travel mode choice (e.g., Bamberg and Schmidt, 2003; Nordlund and Garvill, 2003), while meta-analytical evidence shows personal norms are a consistent and significant predictor of car use (Gardner and Abraham, 2008; Lanzini and Khan, 2017).

In addition to the TPB and NAM, a third line of research focused on the habitual nature of driving, which was neglected by both theories. Habits are defined as “relatively stable behavioral patterns, which have been reinforced in the past […] and are executed without deliberate consideration” (Verplanken et al., 1994). As mobility decisions are repeatedly taken under stable context situations, habits can become particularly important in explaining such decisions (Verplanken et al., 1994). For example, when choosing between various alternatives for a particular journey, people rarely consciously deliberate which travel mode to use. They frequently undertake the same journeys under the same situational conditions, over and over, which can result in automatic behavioral patterns. Empirical studies found that car use habits were a significant predictor of car use behavior (e.g., Bamberg and Schmidt, 2003; Eriksson et al., 2008) and significantly increased the explained variance in travel mode choice, over the explanatory power of rational decision processes (e.g., Verplanken et al., 1994, 1998).

#### Contextual Factors

Contextual variables such as the weather can significantly impact mobility decisions. However, there are only few studies focused on investigating the way individual travel patterns are influenced by the variability in weather conditions (Liu et al., 2015). Two of the most investigated weather aspects are *temperature* and the level of *precipitations*. Studying the impact of temperature, Bergström and Magnusson (2003) found that the number of car trips increased by 27% from summer to winter while the number of bicycle trips decreased by 47%. Research in the United Kingdom also showed that increases in temperature resulted in higher levels of cycling (Parkin et al., 2008), while in Germany, Müller et al. (2008) found a threefold increase in cycling in summer compared to winter. Nevertheless, as previous findings were revealed in places with moderate climates, it remains unclear how ambient temperature affects people’s choice of transportation mode in warmer climates, where increases in temperature during the summer months might make bicycle transportation less feasible. One possible hint is offered by Miranda-Moreno and Nosal’s (2011) study, which showed, on a Canadian sample, that cycling does not linearly increase with temperature and that temperatures above 28°C can actually have a detrimental effect on cycling.

On the other hand, the level of precipitation is frequently mentioned as the most negative weather aspect (Brandenburg et al., 2004) and a reason not to cycle. However, research findings concerning the impact of precipitations on travel mode choice have revealed conflicting results. While some authors found an increase in precipitation determines a switch from active transportation to public transport and private cars (Sabir et al., 2008, 2010; Saneinejad et al., 2012) others found that car traffic is reduced with rainfall (Hassan and Barker, 1999; Keay and Simmonds, 2005). However, according to a recent review investigating the impact of weather on individual travel behavior, warm and dry weather positively impact active transport modes, while rain, snow, wind, cold and hot weather determine a switch from active to sheltered transport modes, such as the car (Böcker et al., 2013).

Another contextual factor that may influence transportation mode is related to the *distance* between the place of origin and the destination. While some people live in places that are too remote from daily necessities to be able to travel in a non-motorized manner, others live so close that walking or cycling are viable travel alternatives. Distance can therefore directly influence the viability of different transportation modes as well as travel decisions (e.g., Schlossberg et al., 2006; Ding et al., 2017).

### The Present Study

The present study aims to contribute to the discussion about a more comprehensive understanding of car use behavior by integrating these three different perspectives (i.e., structural, psychological and contextual) into a single model, in order to investigate how such factors interrelate in predicting car use behavior.

**FIGURE 1:**
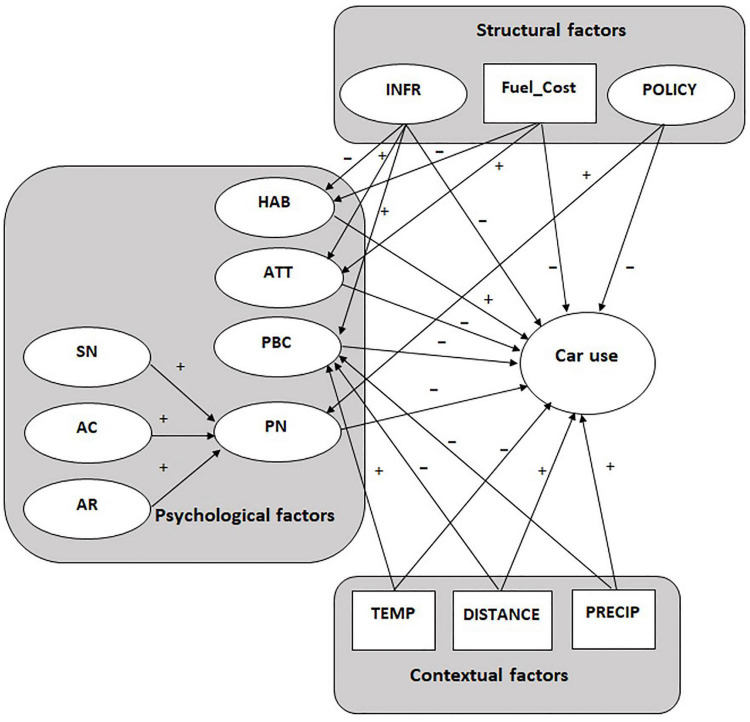
The specified integrative model.

From a structural perspective, previously discussed studies showed that *policies* against excessive car use, the *price of fossil fuel* and available *infrastructure* for transportation alternatives were associated with a decrease in car use. Therefore, we expect that, in the integrated model, stronger policies against car use, higher cost of fuel and better infrastructure for travel alternatives will negatively predict car use. Structural conditions may influence traveling patterns both directly and indirectly, through psychological variables, which are generally more proximal to behavior (Noblet et al., 2014). For example, an improved cycling or public transport infrastructure can also increase people’s perceived control over the use of transportation alternatives as well as improve people’s attitudes toward car use reduction and weaken their car use habits. Therefore, we expect that, in the integrated model, *perceived behavioral control*, *habits* and *attitudes* will mediate the relation between *infrastructure* and *car use.* Similarly, a raised fuel cost may negatively impact people’s attitudes about car transportation and, conversely, improve their attitudes toward car use reduction. At the same time, a high fuel price might stimulate more deliberative decision-making about transportation options, negatively impacting the formation of car use habits. Thus, we expect that *attitudes* and *habits* will mediate the relation between *fuel cost* and *car use*. Concerning psychological predictors, as predicted by the NAM, we expect that *personal norms* related to car transportation will negatively predict car use and will mediate the relation between *awareness of consequences* and *ascription of responsibility*, on the one hand, and car use behavior, on the other. As stipulated by the TPB model, we expect that *attitudes* toward car use reduction and *perceived behavioral control* to reduce car use will negatively predict car use behavior. Because personal moral standards originate also from internalized social norms, some authors argue that personal norms are, at least partly, internalized expectations from important others, be them individuals or authorities (e.g., Harland et al., 1999; Klöckner and Matthies, 2009; Klöckner and Blöbaum, 2010; Liu et al., 2017). Several studies support this claim by showing that personal norms mediate the relation between subjective norms and behavioral intentions or actual environmental behavior (e.g., Hunecke et al., 2001; Liu et al., 2017). Moreover, when personal norms are investigated in the same model with TPB constructs, the direct effect of subjective norms decreases considerably and even becomes non-significant (see Liu et al., 2017; Semenescu and Gavreliuc, 2019). Therefore, in the integrated model, we expect that *personal norms* will mediate the relation between *subjective norms* and authorities’ *policies*, on the one hand, and car use behavior, on the other. As previously discussed, habits directly influence travel mode choice, thus we expect that car use habits will positively predict car use behavior. Concerning contextual predictors, we expect both a direct influence on car use as well as an indirect one, mediated by psychological variables. According to previously discussed studies, we expect that *temperature* will negatively predict car use, while the level of *precipitations* and *distance* to the main travel destination will positively predict it. Adverse and stable weather conditions, or living remotely from work, university or other necessities, can determine beliefs about the unfeasibility of non-motorized means of transportation in such a way that people may believe they lack control over their travel options. Therefore, we expect also an indirect impact of *temperature*, *precipitation* and *distance* on car use, mediated by *perceived behavioral control* (see Figure 1 for a visual representation of all expected relations in the proposed model).

## Materials and Methods

### Participants and Procedure

An ethics approval was obtained from the Ethics Committee of the West University of Timisoara (registration number 23313/0-1/07.06.2019) before the data collection process started. For participating in present study, all participants had to sign an informed consent form, after which they were directed to an online questionnaire. To recruit participants, online announcements, targeted advertising on Facebook, postings on international Facebook groups and the snowball method were used. As an incentive, 10 shopping vouchers worth 25$ each were offered through a random draw. To be eligible for the study, all participants were required to own a driver’s license and have access to a car at any given moment. A total of 619 people completed the questionnaire. Of these, 205 participants were removed, based on the following reasons: 177 did not own a car or have access to one at all times, eighteen did not correctly answer the two test items used in the questionnaire, six observations were duplicate entries, three participants were under 18 years old and one owned an electric car. Participants who owned electric cars were removed, because such drivers may not experience the same motivations for reducing their car use as drivers of cars with conventional fuels. The final sample consisted, therefore, of 414 drivers, living in 47 different countries and on six continents: 0.2% lived in South America, 0.7% in Africa, 1.9% in Australia, 3.6% in Asia, 12.1% in North America and 81.4% in Europe. Within Europe, most of the participants lived in Eastern Europe (67.8%), followed by Western Europe (16.7%), Southern Europe (12.5%) and Northern Europe (3%). Only 9 participants (0.02%) lived in lower-middle income countries, while the rest lived in upper-middle and high income countries, as defined by the World Bank (2021). Participants ranged in age from 18 to 82 (*M* = 32.45, *SD* = 9.83) and were 66.2% female. Of them, 67.9% also owned a bicycle or had access to one at any moment in time, while the average distance, in kilometers, to their most common destination (e.g., work, school, university, etc.) was *M* = 15.72, *SD* = 20.32 (see Table 1). Regarding their car use, 2.7% stated that they never use their car, 13.8% use it rarely, 20.5% use it sometimes, 13.3% use it often, 17.6% very often and 32.1% stated they use it daily.

**TABLE 1:** Demographic characteristics.

Characteristic	
Age, mean (SD)	32.45 (9.83)
Gender	33.8% male
	66.2% female
Continent	0.2% South America
	0.7% Africa
	1.9% Australia
	3.6% Asia
	12.1% North America
	81.4% Europe
Bicycle owners	67.9%
Distance to most common destination, mean (SD)	15.72 (20.33)

### Measures

*Car use* was the dependent variable and was measured with a 6-point scale, by asking participants how often they use their car as a means of transportation (0 = never, 1 = rarely, 2 = sometimes, 3 = often, 4 = very often, 5 = daily).

#### Psychological Variables

*Attitudes (ATT)* toward car use reduction were measured with five semantic differentials, each on a 7-point scale. Participants had to rate to what extent reducing their car use is unattractive/attractive, bad/good, harmful/beneficial, unpleasant/pleasant and unworthy/valuable. Cronbach’s alpha of the scale was, α = 0.89. All items of the scales used can be found in Supplementary Appendix Table 1A.

*Subjective norms (SN)* related to car use reduction were assessed with four items (e.g., “Most people who are important to me would support me in using the car less,” “Most people who are important to me think that I should reduce car transport,” etc.), adapted from Bamberg et al. (2003) and measured on a 7-point scale (1 = totally disagree, 7 = totally agree). Cronbach’s alpha was, α = 0.66.

*Perceived behavioral control (PBC)* to reduce car use was assessed with a 7-point scale used by Bamberg et al. (2003). Participants had to respond to the following two items: “For me to reduce my car use in the future would be” (1 = difficult, 7 = easy) and “My freedom to reduce my car use in the future is” (1 = low, 7 = high). Cronbach’s alpha was, α = 0.86.

*Awareness of consequences (AC)* of car use was assessed with a scale used by Ünal et al. (2018). Participants rated their agreement (1 = totally disagree, 7 = totally agree) with the seven items of the scale (e.g., “The greenhouse effect resulting from road traffic is a serious problem,” “Air pollution resulting from car traffic is a serious problem,” “I am concerned about CO2 emissions resulting from road traffic,” etc.). Cronbach’s alpha of the scale was, α = 0.96.

*Ascription of responsibility (AR)* for negative consequences resulting from car use was measured with a scale composed of three items adapted from Jakovcevic and Steg (2013). Participants rated their agreement or disagreement (1 = totally disagree, 7 = totally agree) with the following items: “I am jointly responsible for the problems caused by car use,” “Not just others, like the government, are responsible for heavy traffic, but me too” and “I feel joint responsibility for the contribution of car traffic to global warming.” Cronbach’s alpha of the scale was, α = 0.89.

*Personal norms (PN)* related to car use reduction were assessed with a scale used by Jakovcevic and Steg (2013). Participants rated their agreement (1 = totally disagree, 7 = totally agree) with the eight items of the scale (e.g., “I feel personally obliged to travel in an environmentally sound way, such as by using a bicycle or public transport,” “I feel obliged to take the environmental consequences of car use into account when making travel choices,” etc.). Cronbach’s alpha was, α = 0.88.

Car use *habits (HAB)* were assessed with the self-report index of habit strength (SRHI, Verplanken and Orbell, 2003). Respondents rated on a 5-point scale (1 = strongly disagree, 5 = strongly agree) their agreement with the 12 items of the scale (e.g., “Using the car is something I do automatically,” “Using the car is something that belongs to my everyday routine,” etc.). Cronbach’s alpha was, α = 0.95.

#### Structural Variables

*Infrastructure (INFR)* for transportation alternatives was measured with three items. Participants rated their agreement (1 = totally disagree, 7 = totally agree) with the following statements: “The transport infrastructure in the place where I live allows me to travel with other means than the car”, “Where I live there are other viable travel alternatives besides the car” and “If I wanted to, I could travel with other means of transportation besides the car.” Cronbach’s alpha of the scale was, α = 0.90.

*Local policies (POLICY)* for car use reduction were assessed with three items, measured on a 7-point scale (1 = totally disagree, 7 = totally agree). Participants rated their agreement with the statements: “Where I live, local authorities encourage sustainable transportation,” “Where I live, local authorities see excessive car transportation as a problem” and “Where I live, local authorities try to reduce private car use.” Cronbach’s alpha of the scale was, α = 0.86.

*Fuel_Cost* was calculated as the ratio between the average price of fuel per country, which was retrieved from GlobalPetrolPrices.com database on 14.02.2020 (about two weeks after the data collection period ended) and the adjusted net national income per capita (World Bank, 2021). Therefore, the higher was the ratio, the higher was the subjective cost for each individual.

#### Contextual Variables

Average annual *temperature (TEMP)* and average amount of yearly *precipitation (PRECIP)* for each location were coded by taking account of participants’ self-reported residential location (i.e., the reported name of city, town or village where they lived) and by using Weather-Atlas.com and Climate-Data.org databases, from where the temperature and precipitation values were retrieved for each particular location.

*Distance* to the main travel destination was measured by asking participants how long (in kilometers) is the distance to their most common travel destination (e.g., university, work, etc.).

#### Statistical Analysis

**FIGURE 2:**
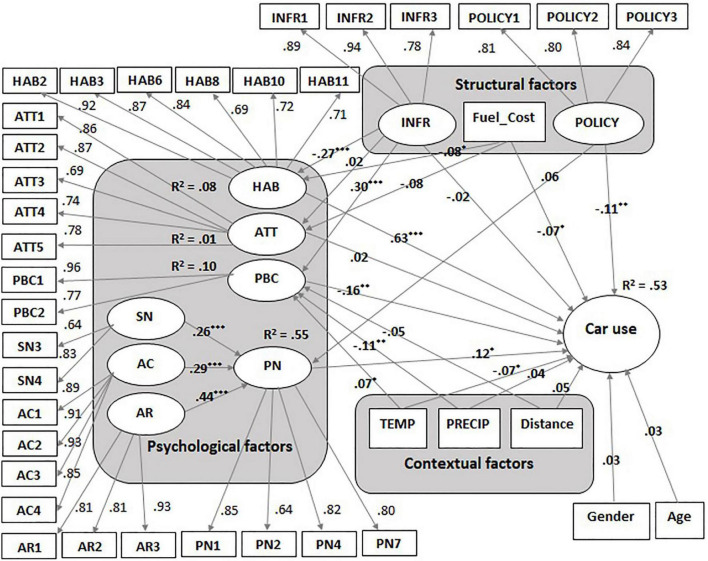
Structural model with standardized path coefficients and explained variance.

A structural equation modeling (SEM) approach was used to conduct the analysis (see Figure 2). Power analysis indicated that for a model with nine latent and four observed variables with an anticipated medium effect size of 0.3, a minimum of 184 participants are needed to detect the effect. In an initial exploratory factor analysis (EFA), convergent and discriminant validity of the nine-factor structure were checked. Next, the measurement model for the latent factors was tested in a confirmatory factor analysis (CFA). After a good fit of the measurement model was obtained, the hypothesized paths in the structural model were tested.

## Results

### Preliminary Analyses

IBM SPSS v.22 and IBM Amos v.22 were used to conduct all the analyses. There were no missing values, except for 139 entries on the three indicators of *POLICY* and 144 on the variable *distance*. An inspection of the missing data revealed no pattern, therefore missing data was imputed using a regression approach. Unengaged responding was checked by inspecting the standard deviations across all responses, for each individual participant. No obvious cases of unengaged responding were revealed.

Next, an exploratory factor analysis (EFA) was conducted on all indicators of latent variables. Kaiser-Meyer-Olkin measure of sampling adequacy was good (*KMO* = 0.91, *p* < 0.001), indicating that the data is suitable for factor analysis. Principal axis factoring with Promax rotation was used as the extraction method, resulting in nine factors with eigenvalues higher than 1, which explained together about 65% of the variability (the pattern matrix can be found in Supplementary Appendix Table 2A). Because of low loadings on factors, indicators SN1, SN2, PN3, PN6, HAB5, HAB9, and HAB12 were removed, to improve the factor structure (all the removed indicators are marked with an asterisk in Supplementary Appendix Table 1A).

### Confirmatory Factor Analysis

Next, in a CFA, the indicators AC5, AC6, AC7, HAB1, HAB4, HAB7, PN5, and PN8 were removed, because of highly correlated errors with other indicators belonging to the same latent variable, which affected model fit. Nevertheless, these indicators were part of large latent reflective factors and were therefore to some extent redundant. The nine-factor measurement model showed a good fit of the data, χ*^2^* = 873.21, df = 428, χ*^2^*/df = 2.04, RMSEA = 0.050 (0.045, 0.055), CFI = 0.95, SRMR = 0.05, PCLOSE = 0.47.

Normality was checked for all indicators of latent factors as well as for all observed variables. All skewness values were between −3 and 3, while in terms of kurtosis only two indicators (AC3 and AC4) and two observed variables (*TEMP* and *PRECIP*) had kurtosis values higher than 3. Nevertheless, with the exception of *PRECIP*, all other kurtosis values fulfilled a more relaxed criteria proposed by Hair et al. (2010), who proposed that skewness values between −3 and 3 an kurtosis values between −7 and 7 are still consistent with the assumption of normality. Therefore, we decided to do a square root transformation for the variable *PRECIP*, to be able to normalize its distribution and retain it in the model.

A final validity check showed good convergent validity for the nine factors (indicated by AVE values close to or higher than.5), good discriminant validity (indicated by square root values of AVE higher than the correlations between factors) and good reliability of the measures (as indicated by Cronbach’s alpha values higher than 0.7; see Table 2).

**TABLE 2:** Validity measures and factor correlation matrix.

	Alpha	AVE	HAB	AC	ATT	PN	INFR	AR	POLICY	SN	PBC
**HAB**	0.92	0.65	0.81								
**AC**	0.94	0.80	−0.17**	0.89							
**ATT**	0.89	0.62	−0.25***	0.31***	0.79						
**PN**	0.87	0.62	−0.50***	0.58***	0.43***	0.79					
**INFR**	0.90	0.76	−0.37***	0.16**	0.08	0.24***	0.87				
**AR**	0.89	0.73	−0.12*	0.57***	0.29***	0.64***	0.15**	0.85			
**POLICY**	0.86	0.67	−0.16**	–0.07	–0.03	0.09	0.43***	0.01	0.82		
**SN**	0.73	0.59	−0.38***	0.20**	0.10	0.36***	0.27***	0.13*	0.19**	0.77	
**PBC**	0.87	0.77	−0.65***	0.23***	0.37***	0.52***	0.39***	0.24***	0.13*	0.34***	0.88

*HAB, car use habits; AC, awareness of consequences; ATT, attitudes toward car use reduction; PN, personal norms for car use reduction; INFR, infrastructure for transportation alternatives; AR, ascription of responsibility; POLICY, local policies against excessive car use; SN, subjective norms for car use reduction; PBC, perceived behavioral control to reduce car use; Alpha, Cronbach’s alpha; AVE, average variance extracted; The square root values of AVE are positioned on the diagonal, while correlation coefficients between factors are placed in non-diagonal positions.*

*p < 0.05,

**p < 0.01,

***p < 0.001.

### Structural Equation Modeling

Imputed values for all factors were created, in order to check for multivariate assumptions. Variable inflation factors (VIF) were investigated for all predictors on the DV. With the exception of PN (VIF = 4.297), all other predictors had VIF values smaller than 2.5, indicating that, aside from PN, there were no serious multicollinearity problems. Nevertheless, PN was kept in the structural model, because it was an endogenous variable. Multivariate influential points were also examined, by studying Cook’s distances. No values greater than.04 were observed, indicating that there were no multivariate influential points. Table 3 includes the correlations between all the variables included in the structural model.

**TABLE 3:** Intercorrelations between the variables included in the model.

	(1)	(2)	(3)	(4)	(5)	(6)	(7)	(8)	(9)	(10)	(11)	(12)	(13)	(14)	(15)	(16)
Car Use (1)	1															
HAB (2)	0.74**	1														
ATT (3)	−0.16**	−0.27**	1													
SN (4)	−0.35**	−0.45**	0.12*	1												
PBC (5)	−0.57**	−0.69**	0.41**	0.41**	1											
AR (6)	0.04	−0.12*	0.32**	0.15**	0.26**	1										
AC (7)	–0.04	−0.18**	0.33**	0.24**	0.24**	0.61**	1									
PN (8)	−0.32**	−0.53**	0.47**	0.45**	0.55**	0.69**	0.63**	1								
INFR (9)	−0.35**	−0.38**	0.08	0.23**	0.41**	0.16**	0.15**	0.23**	1							
POLICY (10)	−0.23**	−0.18**	–0.01	0.21**	0.15**	0.02	–0.08	0.10*	0.48**	1						
PRECIP (11)	0.15**	0.18**	0.10	–0.06	−0.18**	0.01	0.01	–0.07	−0.13**	–0.04	1					
TEMP (12)	0.00	0.09	0.00	–00	–0.04	0.01	–0.01	–0.03	−0.16**	−0.15**	0.37**	1				
Fuel Cost (13)	−0.25**	−0.24**	0.03	0.17**	0.25**	0.16**	0.09	0.19**	0.15**	−0.10*	0.03	0.34**	1			
Distance (14)	0.07	0.03	−0.10*	–0.05	–0.09	–0.01	−0.13*	–0.04	–0.08	0.01	0.04	0.09	0.04	1		
Age (15)	0.08	0.03	0.02	0.02	–0.07	0.00	0.02	0.04	−0.18**	–0.06	–0.04	0.01	−0.11*	–0.05	1	
Gender (16)	–0.06	−0.11*	0.03	0.07	0.11*	–0.10	–0.05	0.00	0.01	–0.01	–0.09	0.01	0.02	0.01	0.07	1

*Notes: HAB = car use habits; ATT = attitudes toward car use reduction; SN = subjective norms for car use reduction; PBC = perceived behavioral control to reduce car use; AR = ascription of responsibility; AC = awareness of consequences; PN = personal norms for car use reduction; INFR = infrastructure for transportation alternatives; POLICY = local policies against excessive car use; PRECIP = square root of annual average precipitations; TEMP = average annual temperature;*

*p < 0.05;

**p < 0.01.

#### Testing the Structural Model

The paths in the structural model were specified according to our hypotheses. The following variables were covaried: HAB was covaried with ATT, PBC, SN, AC, AR, and PN; ATT was covaried with PBC, SN, AR, AC, and PN; SN was covaried with PBC, AC, AR, and POLICY; PBC was covaried with AC, AR, and PN; AC was covaried with AR and POLICY; AR was covaried with INFR and POLICY; INFR was covaried with POLICY and TEMP was covaried with PRECIP (see Table 4). We also controlled for age and gender of participants, because studies showed that such demographic variables are relevant for travel behavior (see Giuliano and Dargay, 2006).

**TABLE 4:** Specified covariance in the structural model.

			Estimate	*SE*	CR	*p*
AC	↔	AR	0.84	0.10	8.68	<0.001
INFR	↔	POLICY	1.25	0.18	7.03	<0.001
SN	↔	AC	0.29	0.09	3.18	0.001
SN	↔	AR	0.17	0.09	1.85	0.064
INFR	↔	AR	0.16	0.11	1.47	0.141
AR	↔	POLICY	–0.01	0.10	–0.05	0.963
AC	↔	POLICY	–0.21	0.09	–2.40	0.016
SN	↔	POLICY	0.15	0.10	1.51	0.130
TEMP	↔	PRECIP	7.05	1.01	6.99	<0.001
PBC	↔	SN	0.66	0.15	4.31	<0.001
PBC	↔	AC	0.42	0.11	3.64	<0.001
PBC	↔	AR	0.48	0.13	3.79	<0.001
PBC	↔	HAB	–0.95	0.11	–8.55	<0.001
HAB	↔	SN	–0.38	0.08	–4.58	<0.001
PN	↔	HAB	–0.43	0.07	–6.38	<0.001
HAB	↔	AC	–0.15	0.06	–2.58	0.010
HAB	↔	AR	–0.08	0.06	–1.23	0.220
PN	↔	PBC	0.73	0.12	5.97	<0.001
HAB	↔	ATT	–0.39	0.09	–4.41	<0.001
PBC	↔	ATT	1.24	0.18	6.91	<0.001
ATT	↔	SN	0.22	0.13	1.79	0.074
ATT	↔	AC	0.61	0.11	5.39	<0.001
PN	↔	ATT	0.52	0.11	4.65	<0.001
ATT	↔	AR	0.65	0.13	5.19	<0.001

*HAB, car use habits; ATT = attitudes toward car use reduction; SN, subjective norms for car use reduction; PBC = perceived behavioral control to reduce car use; AR, ascription of responsibility; AC, awareness of consequences; PN, personal norms for car use reduction; INFR, infrastructure for transportation alternatives; POLICY, local policies against excessive car use; PRECIP, square root of annual average precipitations; TEMP, average annual temperature.*

The fit indices for the structural model showed a good fit, χ*^2^* = 1440.59, df = 661, χ*^2^*/df = 2.18, RMSEA = 0.053 (0.050, 0.057), CFI = 0.92, SRMR = 0.07, PCLOSE = 0.07. As predicted, *car use* behavior was positively predicted by *HAB* (β = 0.63, *t* = 10.53, *p* < 0.001), indicating that stronger car use habits determine higher levels of car use, and was negatively predicted by *PBC* (β = −0.16, *t* = −2.95, *p* = 0.001), *TEMP* (β = −0.07, *t* = −1.88, *p* = 0.031), *Fuel_Cost* (β = −0.07, *t* = −1.95, *p* = 0.026), and *POLICY* (β = −0.11, *t* = −2.47, *p* = 0.007), indicating that stronger perceived behavioral control to reduce car use, higher annual temperatures, a higher relative fuel cost and stronger perceived local policies against excessive car use were associated with less frequent car use. On the other hand, *ATT* (β = 0.02, *t* = 0.50, *p* = 0.691), *distance* (β = 0.05, *t* = 1.38, *p* = 0.083), *PRECIP* (β = 0.04, *t* = 0.92, *p* = 0.179) and *INFR* (β = −0.02, *t* = −0.35, *p* = 0.363) did not have significant direct effects on *car use*, as hypothesized, indicating that attitudes toward car use reduction, distance to the most common travel destination, fuel cost, the level of precipitations and available infrastructure for transportation alternatives did not directly influence people’s car use behavior. A surprising finding was that *PN* positively predicted *car use* (β = 0.12, *t* = 2.40, *p* = 0.016) indicating that the stronger personal norms toward car use reduction are, the more people use their car for transportation. These predictors explained approximately 53% of the variability in car use behavior (*R*^2^ = 0.53).

As expected *PN* was predicted by *AR* (β = 0.44, *t* = 8.05, *p* < 0.001), *AC* (β = 0.29, *t* = 5.36, *p* < 0.001) and *SN* (β = 0.26, *t* = 5.05, *p* < 0.001), but not by *POLICY* (β = 0.06, *t* = 0.1.53, *p* = 0.063). These predictors explained together approximately 55% of the variance in personal norms (*R*^2^ = 0.55).

*HAB* was predicted by *INFR* (β = −0.27, *t* = −5.65, *p* < 0.001) and *Fuel_Cost* (β = −0.08, *t* = −1.97, *p* = 0.025), as anticipated, while *ATT* was negatively predicted by *Fuel_Cost* (β = −0.08, *t* = −1.68, *p* = 0.047) but not by *INFR* (β = 0.02, *t* = 0.36, *p* = 0.361) as we expected. The two predictors explained about 8% of the variability in car use habits (*R*^2^ = 0.08) and 1% of the variability in attitudes toward car use reduction (*R*^2^ = 0.01).

*PBC* was positively predicted by *INFR* (β = 0.30, *t* = 6.48, *p* < 0.001) and *TEMP* (β = 0.07, *t* = 1.74, *p* = 0.042) and negatively predicted by *PRECIP* (β = −0.11, *t* = −2.59, *p* = 0.005), but it was not predicted by *distance* (β = −0.05, *t* = −1.19, *p* = 0.118), as hypothesized (see Table 5 for all paths in the structural model and their associated effects and *p*-values). These predictors explained together approximately 10% of the variance in perceived behavioral control to reduce car use (*R*^2^ = 0.10).

**TABLE 5:** Path coefficients in the structural model.

Path	*b*	β	*Low*β	*High*β	*p*	*R* ^2^
**Direct effects**						
Car use						0.53
HAB → Car use	1.01	0.63	0.53	0.72	<0.001	
PBC → Car use	–0.13	–0.16	–0.29	–0.06	0.002	
PN → Car use	0.11	0.12	0.01	0.22	0.016	
TEMP → Car use	–0.03	–0.07	–0.15	0.00	0.031	
POLICY → Car use	–0.10	–0.11	–0.20	–0.01	0.007	
Distance → Car use	0.00	05	–0.01	0.11	0.083	
Fuel_Cost → Car use	–1576.07	–0.07	–0.14	0.00	0.026	
PRECIP → Car use	0.01	04	–0.03	0.10	0.179	
ATT → Car use	0.02	0.02	–0.08	0.13	0.691	
INFR → Car use	–0.01	–0.02	–0.12	0.08	0.363	
Gender → Car use	0.09	0.03	–0.05	0.09	0.396	
Age → Car use	0.01	0.03	–0.01	0.10	0.349	
PN						0.55
AR → PN	0.55	0.44	0.32	0.58	<0.001	
AC → PN	0.39	0.29	0.17	0.42	<0.001	
SN → PN	0.35	0.26	0.14	0.37	<0.001	
POLICY → PN	0.07	0.06	–0.03	0.15	0.063	
PBC						0.10
INFR → PBC	0.31	0.30	0.20	0.42	<0.001	
TEMP → PBC	0.03	0.07	0.00	0.14	0.042	
PRECIP → PBC	–0.04	–0.11	–0.18	–0.01	0.005	
Distance → PBC	–0.01	–0.05	–0.13	0.04	0.118	
HAB						0.08
INFR → HAB	–0.14	–0.27	–0.37	–0.17	<0.001	
Fuel_Cost → HAB	–1108.11	–0.08	–0.16	0.03	0.025	
ATT						0.01
INFR → ATT	0.02	0.02	–0.08	0.13	0.361	
Fuel_Cost → ATT	–1989.54	–0.08	–0.18	0.02	0.953	
**Indirect effects**						
INFR → PBC → Car use	–0.04	–0.05			0.003	
TEMP → PBC → Car use	–0.01	–0.01			0.022	
PRECIP → PBC → Car use	0.01	0.02			0.009	
INFR → HAB → Car use	–0.14	–0.17			0.006	

*HAB, car use habits; ATT, attitudes toward car use reduction; SN, subjective norms for car use reduction; PBC, perceived behavioral control to reduce car use; AR, ascription of responsibility; AC, awareness of consequences; PN, personal norms for car use reduction; INFR, infrastructure for transportation alternatives; POLICY, local policies against excessive car use; PRECIP, square root of annual average precipitations; TEMP, average annual temperature; Low β, lower limit of the 95% CI for β; Hi β, upper limit of the 95% CI for β.*

We also verified the indirect effects on car use behavior. *INFR* had two significant indirect effects on car use, which were mediated by *PBC* (β = −0.05, *t* = −2.87, *p* = 0.003) and *HAB* (β = −0.17, *t* = −2.51, *p* = 0.006), while *TEMP* (β = −0.01, *t* = −2.01, *p* = 0.022) and *PRECIP* (β = 0.02, *t* = 2.37, *p* = 0.009) had significant indirect effects through *PBC.* The total effects of each variable on car use as well as on all the other predicted variables in the model are represented in Table 6.

**TABLE 6:** Total effects of the predictors in the model.

	Car use	PBC	PN	ATT	HAB
ATT	0.02				
AC	0.03		0.29		
SN	0.03		0.26		
Age	0.03				
Gender	0.03				
AR	0.05		0.44		
PRECIP	0.05	–0.11			
Distance	0.06	–0.05			
TEMP	–0.08	0.07			
POLICY	–0.10		0.06		
Fuel_Cost	–0.12			–0.08	–0.08
PN	0.12				
PBC	–0.16				
INFR	–0.24	0.30		0.02	–0.27
HAB	0.63				

*HAB, car use habits; ATT, attitudes toward car use reduction; SN = subjective norms for car use reduction; PBC, perceived behavioral control to reduce car use; AR, ascription of responsibility; AC, awareness of consequences; PN, personal norms for car use reduction; INFR, infrastructure for transportation alternatives; POLICY, local policies against excessive car use; PRECIP, square root of annual average precipitations; TEMP, average annual temperature.*

## Discussion

The present study was the first attempt to integrate structural, psychological and contextual predictors of car use into a comprehensive model. More precisely, it integrated three structural variables (i.e., infrastructure for transportation alternatives, price of fuel and local policies against excessive car use), seven psychological variables (i.e., attitudes toward car use reduction, subjective norms for car use reduction, perceived behavioral control to use transportation alternatives, awareness of negative consequences of car use, ascription of responsibility for such consequences, personal norms for car use reduction and car use habits) and three contextual variables (i.e., average annual temperature, level of precipitations and distance to main travel destination), while controlling for age and gender of participants. The principal advantage of such an approach is that it allows the possibility to investigate the impact of each predictor while controlling for all other variables included in the model. This adjusts the path coefficients, compared with the situation when predictors are studied in isolation, and thus it portrays a more realistic picture of the existing relations between predictors and the dependent variable. Model fit indices showed a good fit of the model, which explained a considerable degree of the variation in car use behavior (about 53%).

Our results show that, at least one variable from each category of determinants significantly predicted car use, which reinforces the claim that car use is a complex behavior that needs to be understood by taking account of multiple perspectives. Path coefficients show that the most important determinants are psychological factors, most notably habits (β = *0.63*) and perceived behavioral control (β = −0.16), indicating that car use behavior is strongly influenced both by rational and automatic elements. From an interventionist perspective this can seem encouraging, as most psychological variables are modifiable through targeted interventions. In fact, measures to lessen the impact of car use habits and to strengthen perceived behavioral control to use transportation alternatives have already been implemented with promising results (see Bamberg, 2006; Eriksson et al., 2008; Armitage et al., 2011; Ma et al., 2017). Nonetheless, car use habits and people’s perceived behavioral control to reduce car use do not have their sole origin in people’s subjective perspectives but, as our results also show, they are also influenced by objective factors such as available infrastructure or weather conditions. Unlike our expectations, however, attitudes did not significantly predict actual behavior. When attitudes were studied in larger models with multiple predictors similar results were found also by other authors (e.g., Heath and Gifford, 2002; Noblet et al., 2014), who found the influence of attitudes on behavior to be inconsistent across different models and behavioral outcomes in the domain of travel behavior, This may indicate that attitudes may be a more distal predictor to behavior than the theory of planned behavior suggests. In our model, personal norms for car use reduction positively predicted car use, a result which was contrary to what we expected. However, this surprising result might be partially explained by the multicollinearity problem identified previously, which suggests that personal norms were to some extent redundant in the model, and by a reversed causality between *PN* and *car* use, in such a way that people that drive more also feel a stronger sense of personal obligation to reduce their car use. Nevertheless, investigating an alternative model without personal norms revealed path coefficients that were not statistically different from the ones reported in this paper, evidencing that the inclusion of personal norms in the model did not significantly modify results.

Regarding structural predictors, policies against excessive car use significantly predicted car use behavior (β = −0.11). This suggests that people are influenced by top-down information and programs implemented by their local authorities and adjust their behavior accordingly. Even though the lack of a significant impact of local policies on personal norms indicates that top-down information may not be assimilated into the value-system of individuals, local policies can still extrinsically motivate behavior through the interplay between incentives and sanctions. Nevertheless, as studies consistently show (e.g., Foxx and Schaeffer, 1981), the impact of extrinsic motivators on behavior might not always be long lasting, because people revert to their old travel patterns as soon as disincentives are removed. This is one of the main reasons why hard policy measures must also be complemented by soft interventions, which, on the one hand, can increase their acceptability and, on the other hand, can enhance their long-term efficacy.

Our results revealed that fuel cost also predicted car use behavior. A higher fuel cost was associated with less car use, a result that is concordant with previous research (see Goodwin et al., 2004). This indicates that rational cost-benefit calculations also play a direct role in travel behavior, although its impact is smaller when psychological predictors are also considered (β = −0.12). Concerning the impact of infrastructure, our results revealed that existing infrastructure for transportation alternatives did not directly impact car use, but it had a significant indirect impact, mediated by perceived behavioral control and habit formation. Better infrastructure predicted higher perceived behavioral control to use travel alternatives and weaker car use habits, which, in turn, predicted a decrease in car use. In fact, the impact of infrastructure was, after habits, the strongest predictor of the frequency of car use (β = −0.24). Therefore, investing in alternative travel infrastructure (e.g., better public transportation, cycling lanes, etc.) can have a significant impact on travel decisions by increasing people’s perceived freedom to select among different transportation options and ultimately by increasing their availability to make behavioral changes.

The practical implications of the current findings are important, as they suggest a framework which can be used by policymakers to inform future interventions. Such a comprehensive perspective can make it easier for policymakers and practitioners to select and target all relevant determinants in their intervention strategies. Moreover, possessing *a priori* knowledge about the most important determinants of car use can prevent the waste of precious resources on interventions that target less relevant predictors. Based on the results of the present study, addressing car use habits, promoting people’s perceived behavioral control over the use of transportation alternatives and investing in alternative travel infrastructure can prove to be the best strategies for decreasing car use. These results are consistent with Gardner and Abraham’s (2008) and Lanzini and Khan’s (2017) meta-analyses, who found that, besides behavioral intentions, perceived behavioral control to use transportation alternatives and car use habits were the strongest psychological correlates of car use behavior. However, from an interventionist perspective, a recent review found that interventions focused on habits were the least effective of all psychological interventions investigated (see Semenescu et al., 2020). This disparity between theory and practice, however, can only mean that the true potential of habit-centered interventions still remains to be exploited through better designed interventions.

Even though the effects of the measured structural variables were lower than the effects of psychological ones, our results suggest that policymakers and practitioners also need to account for them in their intervention strategies. Combinations of hard (i.e., structural) and soft (i.e., psychological) interventions proved to be more effective in reducing car use than each of them separately (Richter et al., 2011), because such approaches can change both the physical reality and people’s perceptions of that reality (Noblet et al., 2014).

However, travel choices are not always completely voluntary; they are sometimes contingent on the contextual conditions under which people live, such as outside temperature (β = −0.08) or the level of precipitations in their region (β = 0.05), which our study shows they significantly impact car use behavior. Therefore, it is probable that car reduction interventions conducted under favorable contextual conditions will produce a larger impact compared to interventions conducted under conditions that do not allow for many travel alternatives other than motorized transportation, such as too low or too high temperatures or living in regions with heavy precipitations. It is therefore recommended these factors are considered when devising car reduction measures.

### Limitations and Future Studies

Even though the set of determinants employed in the present study was extensive and predicted most of the variance in car use behavior (approximately 53%), it is clear that structural, psychological and contextual conditions represent a heterogeneous set of factors, which can only be covered selectively in an empirical study. Consequently, there is still a large share of the variability in car use behavior that remains unexplained. Future studies will need to expand our proposed framework and include other factors that were previously identified to have an impact on car use, such as the affective and symbolic value of car ownership (e.g., Steg, 2005; Lois and López-Sáez, 2009). Also, because personal norms had a multicollinearity problem, it is important that the results of the present study are replicated on other samples, to verify if the relationships between variables identified in the present study hold and to investigate PN’s relationship with the other variables in the model.

A second limitation is derived from the way the variables in the present study were measured. With the exception of *Fuel_Cost, PRECIP* and *TEMP*, which were coded based on objective information, all other variables in the model were self-reported. While in the case of psychological variables this is unproblematic, it would be ideal to measure structural and contextual variables independently of people’s perceptions, to be able to separate objective and subjective influences on car use behavior. Also, to increase the confidence in the results of mobility studies, objective measurements of car use, such as GPS or odometer measurements, are also recommended. Finally, the variables *FUEL, PRECIP* and *TEMP* were measured either cross-sectionally or by taking yearly averages and thus their heterogeneity was considerably reduced. It is probable that if these variables were examined in longitudinal designs that account for daily variations in fuel prices or weather conditions, their effects on car use would be higher than the ones reported in the present study.

A third limitation is derived from the sample of participants used, which was highly heterogeneous. As previously mentioned, participants had 47 different nationalities and therefore conclusions for a specific population are impossible to formulate. The sample was also unevenly distributed across these countries, as most participants resided in Eastern Europe (most notably in Romania). However, the inclusion of a heterogeneous sample can also be regarded as a strength of the present study, as structural or cultural factors that might be specific for each particular region are likely attenuated by the inclusion of a more diverse sample, resulting in a picture in which such factors probably play only a small role. Moreover, this can also be regarded as a conservatory approach to studying car use, because a higher variability in the sample is likely to result in smaller estimates of effect sizes when compared to more homogenous samples. Therefore, it is likely the conclusions of the present study will remain valid when replicated on more homogeneous populations.

Finally, the present study discusses car use reduction but it does not explain how this change is achieved. While evaluation studies investigating travel mode shift from car to other modes (e.g., Fujii and Kitamura, 2003; Haq et al., 2008) shed some light about the preferred alternatives, the process of change remains poorly understood, as very few studies (e.g., Geng et al., 2016) focused on this aspect. For instance, what are the personal characteristics of those who switch to active modes or of those who change to other motorized transportation (e.g., carpooling or public transport)? Under what structural and contextual circumstances such travel mode change decisions occur? Understanding this process is essential, so that policymakers are able to provide the necessary opportunities during the intervention process. Also, possessing a clear understanding about the preferred alternatives to car transport for each typology of individuals and also about the specific structural and contextual conditions that facilitate mode switch, will allow for more efficient funding of transportation options.

## Conclusion

The present study contributes to a more comprehensive understanding of car use behavior by reuniting three prominent research directions (i.e., psychological, structural and contextual) in a single predictive model. Results show that psychological factors such as car use habits and perceived behavioral control to reduce car use, structural factors such as alternative travel infrastructure, fuel cost and local policies against excessive car use and contextual factors such as temperature and the level of precipitations significantly impact the frequency of car use. From an interventionist perspective, addressing car use habits, perceived behavioral control to use transportation alternatives and travel infrastructure can prove to be especially fruitful for reducing car use behavior, as these variables had the strongest predictive effects. Our results support the inclusion of both structural and psychological factors in the design of TDM policies and plead for a combined hard-soft approach, while also considering relevant contextual variables.

## Data Availability Statement

The datasets presented in this study can be found in online repositories. The names of the repository/repositories and accession number(s) can be found below: https://doi.org/10.17605/OSF.IO/23E8C.

## Ethics Statement

The studies involving human participants were reviewed and approved by Ethics Committee of the West University of Timisoara, 23313/0-1.07.2019; RCE2019-26. The patients/participants provided their written informed consent to participate in this study.

## Author Contributions

Both authors listed made a substantial and direct contribution in elaborating the study design, collecting the data, performing the statistical analysis, writing the draft manuscript, contributing to manuscript revision, and approved it for publication.

## Conflict of Interest

The authors declare that the research was conducted in the absence of any commercial or financial relationships that could be construed as a potential conflict of interest.

## Publisher’s Note

All claims expressed in this article are solely those of the authors and do not necessarily represent those of their affiliated organizations, or those of the publisher, the editors and the reviewers. Any product that may be evaluated in this article, or claim that may be made by its manufacturer, is not guaranteed or endorsed by the publisher.
