# Supplementary material for: Structural, Psychological and Contextual Predictors of Car Use
Source: Front Psychol. 2021 Nov 30;12:692435. doi: 10.3389/fpsyg.2021.692435 (PMC8668941; doi:10.3389/fpsyg.2021.692435)
Supplement: Supplementary file 1 [file Table_1.pdf]

Appendix

Table 1A. *Items of the scales used*

**Attitudes -** How would you describe reducing your car use based on the adjectives described below?	
Unattractive – Attractive	ATT 1
Bad – Good	ATT 2
Harmful – Beneficial	ATT 3
Unpleasant – Pleasant	ATT 4
Unworthy – Valuable	ATT 5
**Perceived behavioral control**	
For me to reduce my car use in the future would be (difficult – easy)	PBC 1
My freedom to reduce my car use in the future is (low – high)	PBC 2
**Subjective norms**	
Most people who are important to me would support me in using the car less	SN 1*
Most people who are important to me think that I should reduce car transport	SN 2*
Most people who are important to me use the car only when necessary	SN 3
Most people who are important to me use sustainable means of transportation such as the bicycle, walking or public transportation, most of the time	SN 4
**Awareness of consequences**	
The greenhouse effect resulting from road traffic is a serious problem	AC 1
Air pollution resulting from car traffic is a serious problem	AC 2
The CO2 emissions resulting from road traffic is a serious problem	AC 3
The emission of particulate matter by motor vehicles is a serious problem	AC 4
I am concerned about global warming resulting from road traffic	AC 5*
I am concerned about the emissions of particulate matter resulting from road traffic	AC 6*
I am concerned about CO2 emissions resulting from road traffic	AC 7*
**Ascription of responsibility**	
I am jointly responsible for the problems caused by car use	AR 1
Not just others, like the government, are responsible for heavy traffic, but me too	AR 2
I feel joint responsibility for the contribution of car traffic to global warming	AR 3
**Personal norms**	
I feel personally obliged to travel in an environmentally sound way, such as by using a bicycle or public transport	PN 1
I would be a better person if I used more often other transport modes instead of the car	PN 2
People like me should do whatever they can to minimize their car use	PN 3*
I feel obliged to take the environmental consequences of car use into account when making travel choices	PN 4
I don’t feel guilty when I use the car even though there are other feasible transport alternatives available (reverse coded)	PN 5_Rev*
If I buy a new car, I feel morally obliged to buy an energy-efficient car	PN 6*
I feel morally obliged to use the car as little as possible, regardless of what other people do	PN 7
I don’t feel personally obliged to use the car as little as possible (reverse coded)	PN 8_Rev*

**Habits**	
Using the car is something I do frequently	HAB 1*
Using the car is something I do automatically	HAB 2
Using the car is something I do without having to consciously remember	HAB 3
Not using the car is something that makes me feel weird	HAB 4*
Not using the car is something that would require effort	HAB 5*
Using the car is something that belongs to my everyday routine	HAB 6
Using the car is something I do without thinking	HAB 7*
Using the car is something I do before I realize I'm doing it	HAB 8
Not using the car is something I would find hard	HAB 9*
Using the car is something I have no need to think about doing	HAB 10
Using the car is something that's typically ''me''	HAB 11
Using the car is something I have been doing for a long time	HAB 12*
**Infrastructure**	
The transport infrastructure in the place where I live allows me to travel with other means than the car	INFR 1
Where I live there are other viable travel alternatives besides the car	INFR 2
If I wanted to, I could travel with other means of transportation besides the car	INFR 3
**Local policies**	
Where I live, local authorities encourage sustainable transportation	POLICY 1
Where I live, local authorities see excessive car transportation as a problem	POLICY 2
Where I live, local authorities try to reduce private car use	POLICY 3

Note: *Indicators with an asterisk were removed from the structural model
