# Supplementary material for: Structural, Psychological and Contextual Predictors of Car Use
Source: Front Psychol. 2021 Nov 30;12:692435. doi: 10.3389/fpsyg.2021.692435 (PMC8668941; doi:10.3389/fpsyg.2021.692435)
Supplement: Supplementary file 2 [file Table_2.pdf]

Appendix

Table 2A. *The pattern matrix resulted from the EFA*

	Factor								
	1	2	3	4	5	6	7	8	9
ATT1			.86						
ATT2			.88						
ATT3			.69						
ATT4			.69						
ATT5			.78						
SN1								.50	
SN2				.36				.43	
SN3								.74	
SN4								.51	
PBC1	-.42								.61
PBC2									.66
AC1		.84							
AC2		.88							
AC3		.96							
AC4		.90							
AC5		.80							
AC6		.84							
AC7		.88							
AR1						.83			
AR2						.85			
AR3						.83			
PN1				.58					
PN2				.60					
PN3				.53					
PN4				.66					
PN5_Rev				.58					
PN6				.39					
PN7				.70					
PN8_Rev				.75					
HAB1	.76								
HAB2	.90								
HAB3	.84								
HAB4	.65								
HAB5	.56								
HAB6	.79								
HAB7	.85								
HAB8	.81								
HAB9	.57								

HAB10	.74			
HAB11	.78			
HAB12	.59			
INFR1			.90	
INFR2			.95	
INFR3			.72	
POLICY1				.75
POLICY2				.78
POLICY3				.88

Note: For a better visualization, coefficients with absolute values below .30 were suppressed
